# Supplementary material for: Complex‐centric proteome profiling by SEC‐SWATH‐MS
Source: Mol Syst Biol. 2019 Jan 14;15(1):e8438. doi: 10.15252/msb.20188438 (PMC6346213; doi:10.15252/msb.20188438)
Supplement: Supplementary file 7 — Dataset EV6 [file MSB-15-e8438-s007.zip › feature_plots_bioplex/Q9P2W9.pdf]

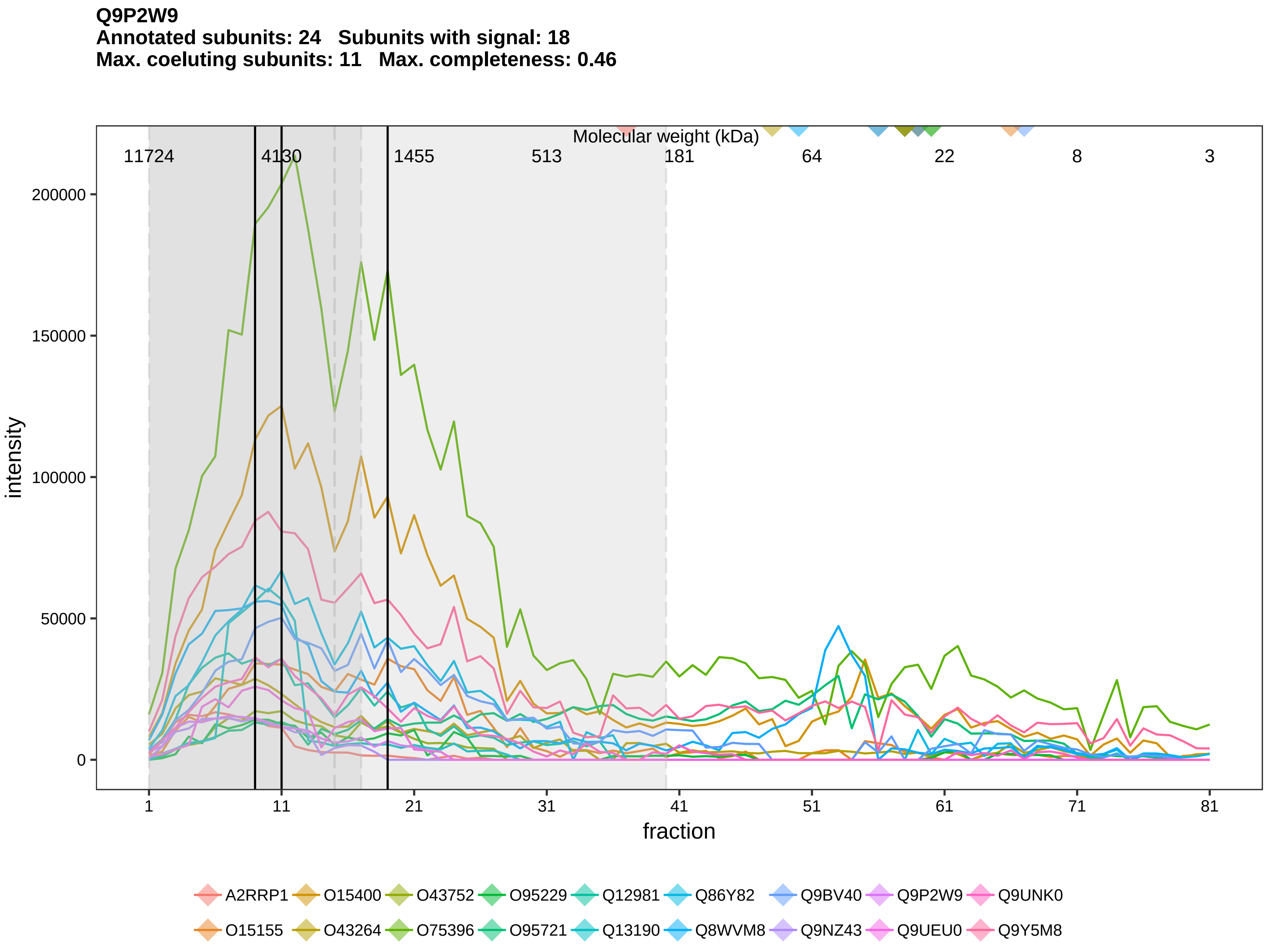
Q9P2W9
Annotated subunits: 24 Subunits with signal: 18
Max. coeluting subunits: 11 Max. completeness: 0.46
Molecular weight (kDa)
11724
4130
1455
513
181
64
22
8
3
intensity
200000
150000
100000
50000
0
1
11
21
31
41
51
61
71
81
fraction
A2RRP1
O15400
O43752
O95229
Q12981
Q86Y82
Q9BV40
Q9P2W9
Q9UNK0
O15155
O43264
O75396
O95721
Q13190
Q8WVM8
Q9NZ43
Q9UEU0
Q9Y5M8
